# Parent–child-agreement on health-related quality of life and its determinants in patients born with Esophageal Atresia: a Swedish–German cross-sectional study

**DOI:** 10.1186/s13023-021-01748-x

**Published:** 2021-03-06

**Authors:** Stefanie Witt, Michaela Dellenmark-Blom, Susanne Kuckuck, Jens Dingemann, Kate Abrahamsson, Carmen Dingemann, John Eric Chaplin, Benno Ure, Monika Bullinger, Vladimir Gatzinsky, Linus Jönsson, Julia Hannah Quitmann

**Affiliations:** 1 Department of Medical Psychology, University Medical Center Hamburg-Eppendorf, Martinistraße 52 – W26, 20246 Hamburg, Germany; 2 Department of Pediatric Surgery, Queen Silvia Children’s Hospital, Sahlgrenska University Hospital, Drottning Silvias Barn O Ungdomsjukh, Rondvägen 10, 41685 Göteborg, Sweden; 3 Department of Pediatric Surgery, Hannover Medical School and Auf der Bult Children’s Hospital, Carl-Neuberg-Straße 1, 30625 Hannover, Germany; 4 Institute of Clinical Sciences, Department of Pediatrics, Gothenburg University, The Queen Silvia Children’s Hospital, 41686 Gothenburg, Sweden

**Keywords:** Esophageal atresia, Congenital malformation, Rare disease, Health-related quality of life, Parent–child agreement

## Abstract

**Background:**

The aim was to compare parent and child-reported health-related quality of life (HRQOL) of children born with esophageal atresia (EA) and determine factors that affect the level of parent–child agreement.

**Methods:**

We included 63 parent–child dyads of children born with EA aged 8–18 from Germany and Sweden. The generic PedsQL 4.0™ questionnaire and the condition-specific EA QOL questionnaire were used to assess children’s HRQOL from parents' and children’s perspectives. The PedsQL™ Family Impact Module was used to assess parental HRQOL and Family Functioning.

**Results:**

On an individual level, intra-class correlation coefficients indicated strong levels of parent–child agreement (.61–.97). At the group level, the analyses showed no significant differences between the responses of parents and children. When a disagreement occurred, parents were more likely to rate generic HRQOL lower than the children (19–35%) and condition-specific HRQOL higher than the children (17–33%). Findings of the binary logistic regression analyzes showed that the child’s age, gender, and country (Germany vs. Sweden) were significant predictors of parent–child agreement in condition-specific HRQOL. We did not identify any significant variables that explain agreement for the generic HRQOL.

**Conclusion:**

The parent–child agreement is mostly good, suggesting that parent-reports are a reliable source of information. However, discrepancies may occur and can be explained by the child's age, gender, and country (Sweden vs. Germany). Both perspectives are essential sources for treating EA patients and should not be considered right or wrong. Instead, this information broadens the perspective on pediatric EA patients.

## Background

Esophageal atresia (EA) is a rare malformation occurring in 2.4/10 000 births. While survival rates today exceed 90%, long-term esophageal and respiratory sequelae remain common [1]. Although neonatal survival has improved, there is scarce data about the patients’ long-term outcomes, including health-related quality of life (HRQOL) [2].

As a multidimensional construct, HRQOL considers the impact of such rare, chronic conditions on overall health and daily living [3], including physical, emotional, and social functioning. It can be measured using generic instruments and condition-specific instruments [4].

In HRQOL related studies of pediatric patients, parents are often asked to report on their children’s HRQOL [5, 6]. Parent proxy-reports are used when the child is very young or cannot complete the self-report due to illness or cognitive impairments [5, 7]. A child’s self-report should be accompanied by a parent-report [5]. Although parents often lack first-hand information, their reports provide important complementary information about children’s HRQOL [8]. Parents might show more awareness, sensitivity, or tolerance due to the child’s health concerns. Including the parental perspective enables clinicians and researchers to broaden their understanding of the child's well-being rather than verify the young patients' responses [9]. Since most rare diseases are diagnosed in childhood, often prenatal or postnatal, affected children experience their disease long-term. However, parents compare the child's HRQOL with healthy siblings, peers, or their own experiences without a rare, chronic health condition. Parents can report more details on physical and medical aspects, especially in very young children. In contrast, children and adolescents can report aspects of social exclusion or inclusion [10]. Only one study examined differences in children’s self-reports and parent proxy-reports in children with EA, which showed satisfactory agreement but with varying results depending on the investigated HRQOL domain [1, 11].

The reliability of using parent proxy-reports has been questioned [12]. Thus, there is a need for further research on the factors affecting the level of (dis-)agreement between parent proxy-reports and child self-reports [13]. Identifying these factors will help gain a deeper understanding of the difference in perspectives of HRQOL (White-Koning et al. 2007), which may provide useful information for guiding clinical decision-making.

Studies have examined the level of agreement between parent proxy-reports and child self-reports of HRQOL for children with chronic diseases but showed inconsistent results [5, 9, 13, 14]. It should be considered that the severity level of the child’s disease may affect the level of parent–child agreement [15]. Parental factors of well-being [16] and stress [17] are associated with parental perceptions of their child’s HRQOL. Simultaneously, the severity level of the child’s disease is negatively associated with parental HRQOL [16], suggesting that parental well-being may affect how parents rate their children’s HRQOL and thus impact the level of parent–child-agreement.

Since there is currently no consistent knowledge of the direction of the discrepancies and its determinants, we aimed to investigate the level of agreement and directional disagreement between child and parent reports of generic and condition-specific HRQOL in 8–17 year-olds born with EA. Furthermore, we aimed to identify significant predictors (age, gender, and EA severity level) of the parent–child-agreement level. We hypothesize that higher parental HRQOL, better family functioning, and higher levels of EA severity are positively associated with a higher level of parent–child-agreement.

## Methods

### Participants and setting

In order to increase the knowledge on parent–child agreement on HRQOL of children born with EA, we conducted this multicenter study at the Department of Pediatric Surgery, Queen Silvia Children’s Hospital in Gothenburg, Sweden, the Centre of Pediatric Surgery, Hannover Medical School, and the Bult Children’s Hospital, Hannover, and the Department for Medical Psychology, University Medical Center Hamburg-Eppendorf, Germany [1]. The Ethical Review Boards of Gothenburg, Sweden (DNR 958-13), and Hannover, Germany (2936–2015) approved the study. Eligible patients with EA Gross type A-E had been treated at these clinics. Patients were considered for participation when they met the following inclusion criteria: (1) children aged 8–17 years at assessment, (2) sufficient German/Swedish language proficiency. We included children eight years of age or older because the EA-QOL^©^ questionnaire is validated for child-reports 8–17 years [18]. The invited families gave informed consent before study participation. The current analyses considered data from the field-test phase [1], including 63 parent–child-dyads.

## Measures

### The PedsQL 4.0™ generic core module

The PedsQL 4.0 ™ questionnaire is a generic instrument to assess children’s HRQOL via child self-report and parent proxy-report. The questionnaire includes 23 items, assessing Physical, Emotional, Social, and School Functioning, and can be calculated into three summary scores (Total Scores, Physical Health Summary Score, and Psychosocial Health Summary Score). The score range between zero and 100, with higher scores indicating better HRQOL (zero is defined as worst possible HRQOL; 100 is defined as best possible HRQOL) [19].

### *The EA-QOL*^*©*^* questionnaire*

The condition-specific EA-QOL^©^ questionnaire consists of a child- and parent-reported 24-item questionnaire for children aged 8–17 years (domains; Eating, Social Relationships, Body Perception, and Health & Well-being; Total Score). The scores are transformed from zero to 100, with higher scores representing better HRQOL [18].

### PedsQL™ family impact module

The PedsQL™ Family Impact Module (PedsQL™ FIM) was used to assess parental HRQOL and Family Functioning. Parental HRQOL (20 items) was calculated as the mean of items from the domains Physical, Emotional, Social, and Cognitive Functioning. Family Functioning (8 items) was calculated from the subscales Daily Activities and Family Relationships. Scores range from zero to 100, with higher scores representing higher HRQOL, as well as better Family Functioning [20].

We used the measurements mentioned above to assess the HRQOL of children and adolescents born with EA because of their availability in both countries. Furthermore, research on patient-reported outcome measures (PROMs) to assess HRQOL in children with rare, chronic diseases reported the PedsQL generic module as acceptable and appropriate in measuring the needs and experiences of children with rare, chronic health conditions and their parents. Additionally, pediatric patients and their parents also appreciated condition-specific HRQOL measurements focusing on disease-specific daily life challenges [21].

All measurements used for assessing HRQOL from the child- and parent-perspective, including the PedsQL 4.0 ™ questionnaire, the EA-QOL^©^ questionnaire, and the PedsQL™ Family Impact Module, were validated for the included age groups and countries [18, 22, 23].

### Statistical analysis

Parent–child-agreement on children’s HRQOL was examined at the individual and group level [24], using intra-class correlation coefficients [ICC] [25], respectively the Friedman-Test. We entered children’s age, children’s gender, the severity level of EA (mild to moderate vs. severe) [26], parental HRQOL, family functioning, and the country as covariates into the model.

We calculated parent–child-disagreement as absolute and directional discrepancies. Directional discrepancies were categorized into three groups (“parent-report < child-report”, “agreement”, and “parent-report > child-report”) based on the threshold for minimally important differences (MID) in quality of life. The meta-analysis of Norman, Sloan [27] showed that the threshold of discrimination for changes in HRQOL in patients with chronic health conditions appears to be approximately half a standard deviation (SD). Hence, we used this threshold of half an SD to evaluate essential differences between child- and parent-reports of HRQOL.

We performed binary logistic regression analyses to identify variables that predict parent–child-agreement (parent-report < child-report vs. agreement; parent-report > child-report vs. agreement) for the PedsQL 4.0™ total score and the EA-QOL^©^ total score. Children’s gender, children’s age, severity level of EA, parental HRQOL, family functioning, and the country (Germany vs. Sweden) were entered as predictors.

Statistical analyses was performed using SPSS (version 25.0). We replaced missing values by the individual mean score for each variable except for sociodemographic and clinical variables. Missing data was defined as random and with less than 25% of the values (EA-QOL^*©*^) and with less than 50% of the values (PedsQL 4.0 ™). We tested differences of sociodemographic and clinical variables between both samples from Germany and Sweden using the Mann–Whitney U-Test or chi-square test when appropriate.

## Results

Chi-square respectively Mann–Whitney U-Test showed that the children's sociodemographic and clinical characteristics were similarly distributed between German and Swedish participants, except for associated anomalies and anorectal malformations. Parent characteristics showed differences between both countries (Table 1). Furthermore, no significant differences were found between respondents and non-respondents in the EA-QOL study regarding clinical factors [18]. Hence, we assume that the present sample represents typical pediatric EA patients in Germany and Sweden due to clinical variables. Differences between respondents and non-respondents based on parental characteristics and parental commitment cannot be precluded.

**Table 1:** Children’s and parent’s sociodemographic characteristics of the German and Swedish sample

Children’s characteristics	German sample	Swedish sample	z or χ^2^	U or df	p
	n = 23	n = 40			
	No. (%) or M (± SD)	No. (%) or M (± SD)			
Child age (in years)	12.77 (2.98)	12.24 (3.46)	**−** .792	404.50	.43
Child gender					
Male	13 (56.5%)	22 (55%)	.78	1	.38
Female	10 (43.5%)	18 (45%)			
EA severity level^a^					
Mild/moderate	14 (60.9%)	18 (45%)	.02	1	.90
Severe	9 (39.1%)	22 (55%)			
Primary anastomosis					
Yes	21 (95.7%)	34 (85%)	1.68	1	.20
No	2 (4.3%)	6 (15%)			
Associated anomalies					
Yes	15 (65.2%)	29 (72.5%)	9.92	1	.01
No	8 (34.8%)	11 (27.5%)			
Cardiac malformation					
Yes	7 (30.4%)	18 (45%)	2.68	1	.10
No	16 (69.6%)	22 (55%)			
Anorectal malformation					
Yes	5 (21.7%)	5 (12.5%)	29.35	1	≤ .01
No	18 (78.3%)	35 (87.5%)			
Birthweight (in grams)					
≤ 2,500 g	7 (30.4%)^b^	15 (37.5%)^c^	1.85	1	.17
> 2,500 g	9 (39.1%)	23 (57.5%)			
Gestational age (in weeks)	35.65 (3.71)^d^	36.44 (3.61)^e^	**−** .852	284.00	.39
**Parents’ characteristics**	**No. (%) or Mean (± SD)**	**No. (%) or Mean (± SD**)	**z or** **χ**^**2**^	**U or df**	**p**
Parent age	46.04 (7.11)	43.52 (6.33)^e^	**−** 2.138	299.00	.03
Parent gender					
Male	1 (4.3%)	5 (12.5%)^e^	40.32	1	≤ .01
Female	22 (95.7%)	34 (85%)			
Partnership					
Single parent	1 (4.3%)^b^	9 (22.5%)^e^	27.56	1	≤ .01
Co-habitant parent	21 (91.3%)	30 (75%)			
Employment status					
Full/part time worker	18 (78.3%)^e^	36 (90%)^c^	111.97	6	≤ .01
Parental/sick leave /unemployed	4 (17.4%)	2 (5%)			
Health status					
Healthy	18 (78.3%)^e^	33 (82.5%)^e^	27.56	1	≤ .01
Doctor diagnosis	4 (17.4%)	6 (15%)			

M = Mean, SD = Standard deviation, z = z-value, χ^2^ = chi-square, U = Mann–Whitney U, df = degrees of freedom, p = p-value, EA = Esophageal atresia

^a^ The severity of EA was divided into mild/moderate and severe according to predefined clinical criteria published elsewhere [26]

^b^ 7 missings

^c^ 2 missings

^d^ 6 missings

^e^ 1 missing

At the individual level, the ICCs indicated strong levels of agreement between parent- and child-reported children’s HRQOL for both generic and condition-specific HRQOL (Table 2). At the group level, the Friedman-Test showed no significant differences between the responses of parents and children concerning sociodemographic and clinical variables, except for the generic domain School in the German sample (χ^2^_(1)_ = 4.765, p = 0.03, n = 22) (Table 2).

**Table 2:** Inter-rater reliability: analyses of covariance for repeated measures (ANCOVA); country-specific

		Parent-report		Child-report		ICC [95% CI] ^g^	Friedman-Test		
	Dyads n	M (SD)	α	M (SD)	α		χ^2^	df	p
GERMANY PedsQL									
Physical	23	80.84 (20.63)	.86	84.65 (18.89)	.87	.87 [.69, .95]**	.286	1	.59
Emotional	21^a^	79.76 (23.05)	.90	86.43 (17.90)	.84	.82 [.55, .93]**	2.571	1	.11
Social	23	80.87 (21.57)	.82	83.62 (16.84)	.78	.73 [.37, .89]**	2.579	1	.11
School	22^b^	69.77 (19.67)	.78	75.68 (19.60)	.84	.83 [.60, .93]**	4.765	1	.03
Psychosocial	21^a^	77.94 (17.46)	.90	82.46 (14.28)	.88	.79 [.47, .91]**	2.000	1	.16
Total	21^a^	79.32 (16.93)	.93	83.46 (14.08)	.92	.79 [.49, .92]**	1.316	1	.25
SWEDEN PedsQL									
Physical	32^c^	82.32 (23.04)	.91	84.67 (21.64)	.92	.95 [.90, .98]**	1.190	1	.28
Emotional	31^d^	81.67 (22.34)	.90	84.03 (20.47)	.87	.90 [.80, .95]**	.727	1	.39
Social	31^d^	88.06 (17.64)	.85	88.55 (18.45)	.88	.90 [.79, .95]**	.692	1	.41
School	32^c^	77.93 (20.66)	.85	80.31 (19.30)	.83	.95 [.90, .98]**	1.636	1	.20
Psychosocial	31^d^	82.92 (17.56)	.92	84.62 (16.79)	.93	.95 [.90, .98]**	.667	1	.41
Total	31^d^	82.93 (18.55)	.95	84.91 (17.14)	.96	.97 [.93, .98]**	1.960	1	.16
GERMANY EA-QOL									
Eating	21^a^	75.85 (20.36)	.81	72.64 (19.63)	.72	.89 [.72, .95]**	.222	1	.64
Body	20^e^	84.50 (20.38)	.87	85.00 (21.64)	.86	.61 [**−** .01, .85]*	.333	1	.56
Social	21^a^	79.65 (19.80)	.84	76.70 (21.21)	.79	.89 [.74, .96]**	.692	1	.41
Health	20^e^	84.38 (16.78)	.64	80.94 (23.51)	.86	.78 [.46, .91]**	.077	1	.78
Total	20^e^	80.65 (16.69)	.92	78.25 (17.73)	.88	.90 [.76, .96]**	.250	1	.62
SWEDEN EA-QOL									
Eating	36^f^	73.61 (21.36)	.84	71.44 (19.75)	.76	.93 [.97, .97]**	3.846	1	.05
Body	38^a^	80.00 (19.59)	.82	80.39 (20.94)	.81	.95 [.91, .98]**	.182	1	.67
Social	38^a^	78.38 (16.71)	.74	74.53 (17.30)	.74	.86 [.73, .93]**	1.960	1	.16
Health	38^a^	81.41 (17.28)	.70	80.26 (19.25)	.76	.92 [.85, .96]**	.800	1	.37
Total	36^f^	78.18 (15.32)	.91	76.09 (15.55)	.89	.93 [.86, .97]**	2.613	1	.11

M = Mean, SD = Standard deviation, α = Cronbach’s alpha, ICC = Intraclass correlation coefficients, CI = Confidence interval, χ^2^ = Chi-Square, df = degrees of freedom, p = p-value (significance)

^a^ 2 missing dyads

^b^ 1 missing dyad

^c^ 8 missing dyads

^d^ 9 missing dyads

^e^ 3 missing dyads

^f^ 4 missing dyads

^g^ Intraclass correlation coefficients reference values: ICC < .40 = poor agreement, ICC between .41 and .60 = moderate agreement, ICC between .61 and .80 = good agreement, ICC > .81 = excellent agreement [25]. *≤.05; **≤.01

**Fig. 1:**
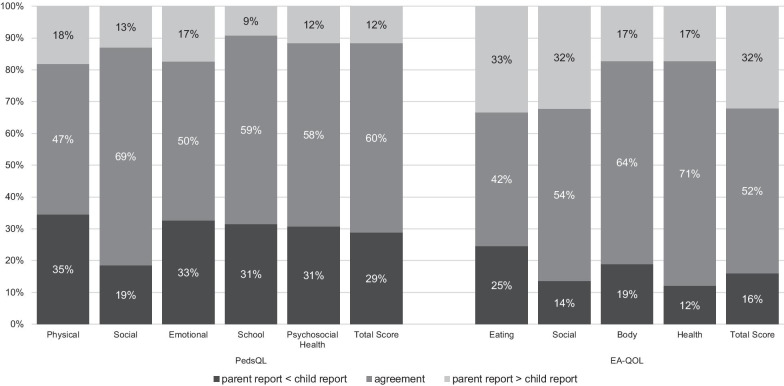
Distribution of parent–child directional discrepancies on reports of generic and condition-specific HRQOL

**Fig. 2:**
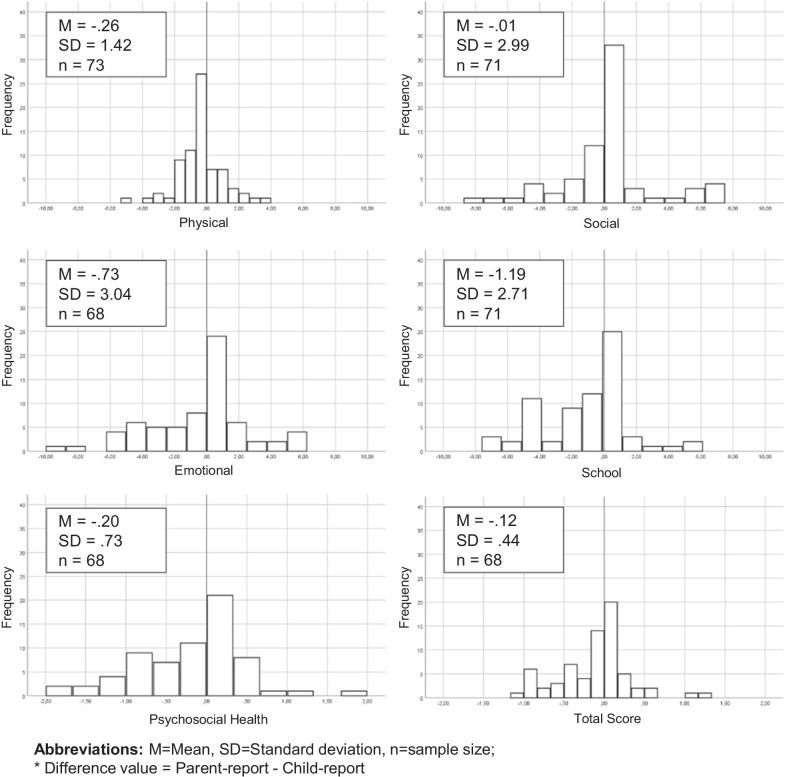
Histograms for difference values* for the PedsQL. M, Mean; SD, Standard deviation; n, sample size; * Difference value = Parent-report - Child-report

**Fig. 3:**
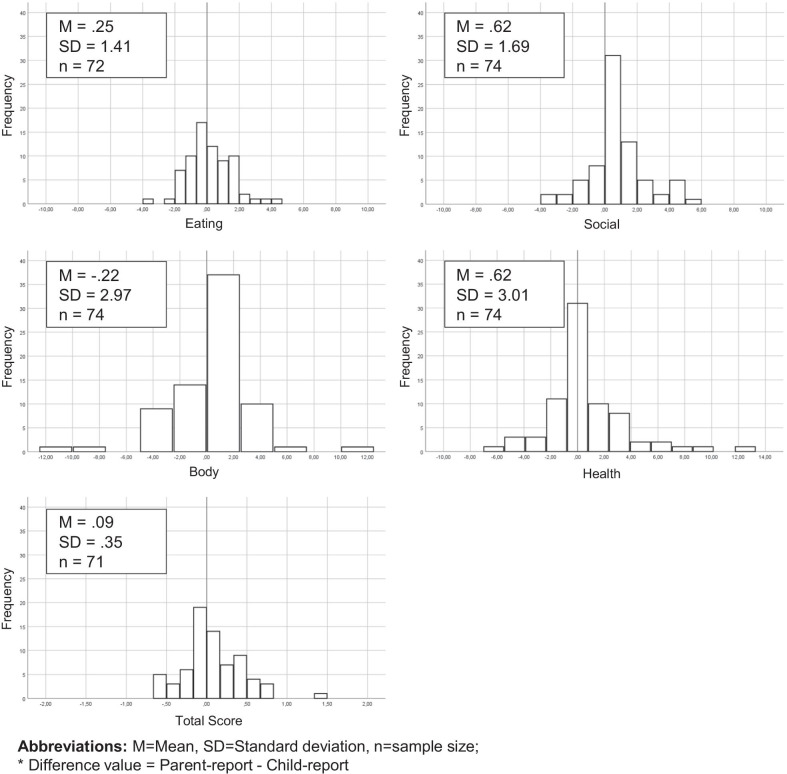
Histograms for difference values for the EA-QOL. M, Mean; SD, Standard deviation; n, sample size; * Difference value = Parent-report - Child-report

The examination of the directional discrepancies between parent- and child-reports demonstrated 47–69% agreement between children and parents in rating generic HRQOL and 42–71% in rating condition-specific HRQOL. In the remaining cases, a disagreement occurred. In the disagreement cases, parents were more likely to rate generic HRQOL lower than the children (19 vs. 35%). At the same time, parents were more likely to rate children’s HRQOL higher than the children when using condition-specific measures (17 vs. 33%) (Fig. 1). Figures 2 and 3 present a detailed distribution of the parent–child difference values for the generic PedsQL 4.0™ and the condition-specific EA-QOL^©^ questionnaire on the domain level and the total scores.

The binary logistic regression analyzes show that both the model as a whole ((χ^2^_(6)_ = 9.887, p = 0.13, n = 51); (χ^2^_(6)_ = 8.016, p = 0.24, n = 45)) and the independent variables did not show any significant effect for the generic PedsQL 4.0™ core module (Table 3). However, the results of the binary logistic regression analyzes for the condition-specific EA-QOL^©^ questionnaire show that whole model ((χ^2^_(6)_ = 15.957, p = 0.01, n = 44); (χ^2^_(6)_ = 14.848, p = 0.02, n = 55)) and some of the independent coefficients of the variables are significant. On the one hand, using the condition-specific *EA-QOL*^*©*^ questionnaire, results showed that child’s age (Wald_(1)_ = 4.46, p = 0.03), child’s gender (Wald_(1)_ = 4.88, p = 0.03), and the origin of the country (Wald_(1)_ = 5.07, p = 0.02) predict the parent–child agreement compared to underratings of children's HRQOL by the parents. If the child is a boy, the relative probability of parent–child disagreement (parents underrating children’s HRQOL vs. agreement) increases by 45%. If the child is one unit younger, the relative probability of parent–child disagreement increases by 34.7%. If the participants are from the German sample, the probability of disagreement increases significantly (Table 4).

**Table 3:** Binary logistic regression analysis in EA patients and their parents using the generic PedsQL core module

	PedsQL total score							
	PR < CR versus agreement^a^ (n = 51)				PR > CR versus agreement^b^ (n = 45)			
	B	Wald	p	[95% CI]	B	Wald	p	[95% CI]
Child gender^c^	**−** 1.12	2.24	.13	[.08, 1.41]	1.57	3.51	.06	[.93, 24.87]
Child age	.02	.03	.87	[.81, 1.29]	.13	1.24	.27	[.91, 1.43]
EA severity level^d^	.59	.56	.45	[.39, 8.36]	.02	.00	.98	[.21, 5.01]
Parental HRQOL	.01	.06	.80	[.95, 1.07]	.00	.01	.92	[.93, 1.08]
Family functioning	**−** .04	2.15	.14	[.91, 1.01]	**−** .04	1.31	.25	[.90, 1.03]
Country^e^	**−** 1.08	2.26	.13	[.08, 1.39]	**−** .56	.44	.51	[.11, 3.00]
Model summary	R^2^ = .24				R^2^ = .23			
	χ^2^(6) = 9.887, p = .13				χ^2^(6) = 8.016, p = .24			

PR = Parent-report, CR = Child-report, B = regression coefficient, Wald = Wald-value, p = p-value, CI = confidence interval, R^2^ = Nagelkerkes R-square, EA = Esophageal atresia, HRQOL = Health-related quality of life

^a^ 0 = agreement, 1 = PR < CR, defined using a threshold of ½ SD

^b^ 0 = agreement, 1 = PR > CR, defined using a threshold of ½ SD

^c^ Child gender: 0 = male, 1 = female

^d^ EA severity level: 0 = mild moderate, 1 = severe (Dellenmark–Blom, Dingemann, Witt, Quitmann, Jönsson et al. 2018)

^e^ Country: 0 = Germany; 1 = Sweden

**Table 4:** Binary logistic regression analysis in EA patients and their parents using the condition-specific EA-QOL questionnaire

	EA QOL total score							
	PR < CR versus agreement^a^ (n = 51)				PR > CR versus agreement^b^ (n = 45)			
	B	Wald	p	[95% CI]	B	Wald	p	[95% CI]
Child gender^c^	**−** **2.89**	**4.46**	**.03**	**[.01,** **.81]**	**1.89**	**6.21**	**.01**	**[1.45,** **29.06]**
Child age	**−** **.43**	**4.88**	**.03**	**[.45,** **.95]**	**.24**	**4.85**	**.03**	**[1.03,** **1.59]**
EA severity level^d^	.29	.08	.78	[.18, 10.02]	**−** .52	.39	.53	[.12, 3.02]
Parental HRQOL	**−** .05	1.21	.27	[.88, 1.04]	**−** .05	2.94	.09	[.90, 1.01]
Family functioning	.05	1.37	.24	[.97, 1.14]	.02	.72	.40	[.97, 1.08]
Country^e^	**2.27**	**5.07**	**.02**	**[1.34,** **69.63]**	.96	1.86	.17	[.66, 10.34]
Model summary	R^2^ = .43				R^2^ = .32			
	χ^2^(6) = 15.957, p = .01				χ^2^(6) = 14.849, p = .02			

PR = Parent-report, CR = Child-report, B = regression coefficient, Wald = Wald-value, p = p-value, CI = confidence interval, R^2^ = Nagelkerkes R-square, EA = Esophageal atresia, HRQOL = Health-related quality of life

^a^ 0 = agreement, 1 = PR < CR, defined using a threshold of ½ SD

^b^ 0 = agreement, 1 = PR > CR, defined using a threshold of ½ SD

^c^ Child gender: 0 = male, 1 = female

^d^ EA severity level: 0 = mild moderate, 1 = severe (Dellenmark–Blom, Dingemann, Witt, Quitmann, Jönsson et al. 2018)

^e^ Country: 0 = Germany; 1 = Sweden

On the other hand, the analysis identified the child’s gender (Wald_(1)_ = 6.21, p = 0.01) and child’s age (Wald_(1)_ = 4.85, p = 0.03) as significant variables predicting parent–child agreement compared to parents’ overrating of children’s HRQOL. In contrast to the analysis of parents’ underratings, the analyzis of parents' overrating shows an opposite direction for the significant variables child's gender and child’s age. If the child is a girl, parents' relative probability of overrating increases by 59.1%. If the child is older, the probability increases by 27.6% (Table 4). We did not confirm our hypotheses regarding the negative association of lower parental HRQOL and lower severity on the parent–child agreement.

## Discussion

This study investigated the parent–child-agreement of HRQOL in a sample of children and adolescents born with EA and the determinants of discrepancies. Inconsistent with previous findings from the literature, which advocate moderate levels of agreement in pediatric HRQOL assessment, we found strong levels of agreement between child- and parent-reported children’s HRQOL on the individual level. While Quitmann, Rohenkohl [13] also reported at least moderate to good ICC levels for children and adolescents with short stature and their parents, other studies reported only moderate levels of parent–child agreement in chronic diseases [8, 28].

Contrary to previous studies' results [13, 29], we found no differences in the agreement between generic and condition-specific instruments. According to our results, the agreement was good between children and parents [1]. However, a proportion of child-parent dyads also demonstrated directional differences in children’s generic and condition-specific HRQOL ratings. Interestingly, the direction of the differences between parent- and child-reported children’s HRQOL differed depending on the instrument used. Parents tended to underrate children’s HRQOL using generic HRQOL measures. Parents of children with rare, chronic diseases often experience their child's diagnosis as a traumatic experience that completely changes their previous life and plans [30]. Furthermore, they tend to compare their child's health and quality of life with healthy peers because of their loss of the ‘perfect’ child [31]. This comparison explains the underrating of the generic HRQOL compared to the perspective of the affected children and adolescents.

Simultaneously, the parents were more likely to score their children’s HRQOL higher than the children when using the condition-specific tool, except the Body Perception domain. A possible explanation relates to the nature of the different measurement levels' questions. A condition-specific instrument is more sensitive to clinical characteristics and raises relevance for the patient group. The EA-QOL^©^ questionnaire was developed using the child's experiences as primary importance and parents’ as complementary importance. Therefore, it might be easier for children to answer those questions [26, 32]. The intensity of the experienced limitation and the most recent experiences are essential for assessing the subjective HRQOL [33, 34]. Since children and adolescents born with EA experience their chronic health condition directly, they might experience condition-specific aspects of HRQOL more burdensome than generic aspects. Hence, children and adolescents rate their HRQOL lower than their parents.

Approximately half of the dyads showed an agreement—defined as differences of equal or less than half of the standard deviation of the score—between the parent’s perspective and the children’s perspective. When disagreement occurred, it was likely to be in the direction of parents underrating children’s HRQOL using the generic HRQOL measurement. Except for the domain Social Functioning, the underrating was present in approximately one-third of parents. The same pattern was found in other studies of children with chronic health conditions [5]. Our results are consistent with previous research in children with chronic diseases, which found that parents tend to underestimate their child’s HRQOL [5, 8, 13, 14, 35, 36]. However, the direction of disagreement we found in our sample for the condition-specific HRQOL measurement was inconsistent with these findings. One-third of parents overrated their children’s HRQOL, especially in the domains Eating, Social Relationships, and the Total Score. Here again, it might be that the questionnaire was more sensitive to the children’s perspective than the parents’ since it was developed primarily according to the children’s experiences [1, 26, 32].

On the domain level, our findings showed that the lowest rates of agreement were present in the generic domain Physical Functioning and the condition-specific domain Eating. Both domains can be regarded as observable HRQOL dimensions for the parents that are not related to the child's internal experiences. The level of agreement was found to be highest in the domain Social Functioning using the PedsQL 4.0™ while using the EA-QOL^©^, the agreement was highest in the domain Health & Wellbeing. These results contrast with previous research, which describes better agreement for observable dimensions [8, 37, 38]. These results underline the importance of capturing the child-report using the EA-QOL^©^ questionnaire in clinical practice when monitoring and providing supportive interventions to the child’s HRQOL.

In our analyses, sociodemographic information (age, gender, country) contributed to explaining a significant variation of the parent–child disagreement, but only for the condition-specific EA-QOL^©^ questionnaire and not for the generic HRQOL. Our results showed that parents underestimate children’s HRQOL when the children are younger and overestimate children’s HRQL when the children are older. We assume that children use their peers to talk about personal stress and burden with increasing age. Parents are no longer close to their children's experiences and may interpret less communication as an improvement in coping. The current literature is inconsistent regarding age's effect on the parent–child agreement. Some studies described higher agreement in younger children supporting the hypothesis that increased independence during puberty may limit the exchange between parents and children [38, 39]. In contrast, other studies found higher levels of agreement for older children attributed to growing cognitive and communication skills [40, 41]. However, some studies did not find a significant influence of child age on the level of parent–child agreement [37]. Nevertheless, only a few studies consider this variable in statistical analysis [9, 42].

Clinical and parental characteristics did not significantly affect the parent–child agreement in EA patients and their parents. Other studies reported that the level of agreement between parents and children seemed more strongly associated with familial and social factors [13, 28, 43]. The residence country explained the underestimating of parents in our study using the condition-specific tool. It is difficult to identify a definite explanation for these results. However, there might be different norms or traditions of how parents and children in different countries communicate about their health condition and its consequences in daily life [44].

Regarding the child’s gender, we showed that being a boy was associated with a higher relative probability of parents’ underestimating children’s HRQOL. Being a girl increases the probability of parents’ overestimating children's HRQOL. In contrast to our results, other studies reported that the child’s gender did not significantly affect the parent–child agreement [45–47]. Waters, Stewart–Brown [48] reported that-parent-female child-dyads showed lower agreement than parent-boy dyads. However, this study only included adolescents. Gender and age are rarely examined in studies and need to be investigated in more detail.

## Limitations

Some limitations should be taken into account when interpreting the present study results. Concerning the sample, it should first be mentioned that the parents’ perspective mostly consisted of mothers’ reports due to fathers' low response rate. Even though this is not uncommon in pediatric health care studies and clinical practice [9], the results are biased towards the mothers’ perspective. They should be replicated in future studies using larger fathers' samples to determine whether there are differences between father and mother reports. Although this sample size is relatively large for a rare disease study such as EA, the small sample size of 63 parent–child-dyads and the different sample composition in Germany and Sweden does not allow us to draw meaningful conclusions. The missing data for single domains must be considered when interpreting the results. Missing data resulted from single items that were not answered by individuals. Since both parents and children's data had to be available in full to compare the perspectives, dyads had to be excluded as soon as the domains could not be calculated for both. This small sample size should also be considered when interpreting the 95% CI.

Moreover, our study sample seems fairly representative regarding gestational age at birth, primary esophageal repair, and child gender. However, the prevalence of associated anomalies seems slightly higher than in previous reports, especially cardio-vascular anomalies [49].

Another limitation concerns the methods of data collection. Since families have filled out the questionnaires at home, a parental influence of the children’s answers cannot be excluded even though parents have explicitly been asked not to influence their children’s answers. This study's results can be used as an indication of future studies investigating parent–child agreement in pediatric patients with rare malformations such as EA.

## Conclusion

Of importance to clinical practice and future research, the parent–child-agreement when rating EA children’s HRQOL is mostly good, suggesting that parent reports are a reliable source of information. However, discrepancies may occur, which may differ in the direction in the child-report depending on generic or condition-specific measurement level. The child's age, gender, and country origin (PR < CR vs. agreement) can explain the parent–child-agreement using condition-specific HRQOL measurements. These variables need to be investigated in larger samples and need to be considered when interpreting HRQOL measures of EA patients and their parents. Nevertheless, the various perspectives are essential sources for treating EA patients and should not be considered right or wrong. Instead, this information broadens the perspective on pediatric EA patients.

## Key messages

- Agreement between child and parent reports when rating EA children’s HRQOL is mainly good, suggesting that parent-reports are a reliable source of information.
- In EA children, the directional discrepancy in parent-report vs. child-report shows parents' tendency to underrate their children’s generic HRQOL and overrate the condition-specific HRQOL.
- In condition-specific HRQOL assessment of EA children, disagreement between child- and parent-report may be explained by the child’s age, gender, and country origin (Sweden vs. Germany).

## Data Availability

The datasets used and/or analyzed during the current study are available from the corresponding author on reasonable request.

## Abbreviations

HRQOL: Health-related quality of life
EA: Esophageal atresia
PedsQL 4.0™: Pediatric quality of life inventory
EA-QOL^©^: The Esophageal-Atresia-quality-of-life
SD: Standard deviation
